# Supplementary material for: Hypoxia-induced metastatic heterogeneity in pancreatic cancer
Source: bioRxiv. 2025 Aug 29:2025.08.26.672389. Preprint. [Version 1] doi: 10.1101/2025.08.26.672389 (PMC12407890; doi:10.1101/2025.08.26.672389)
Supplement: 1 [file NIHPP2025.08.26.672389V1-supplement-1.pdf]

Figure S1

bioRxiv preprint doi: https://doi.org/10.1101/2025.08.26.672389; this version posted August 29, 2025. The copyright holder for this preprint (which was not certified by peer review) is the author/funder, who has granted bioRxiv a license to display the preprint in perpetuity. It is made available under aCC-BY 4.0 International license.

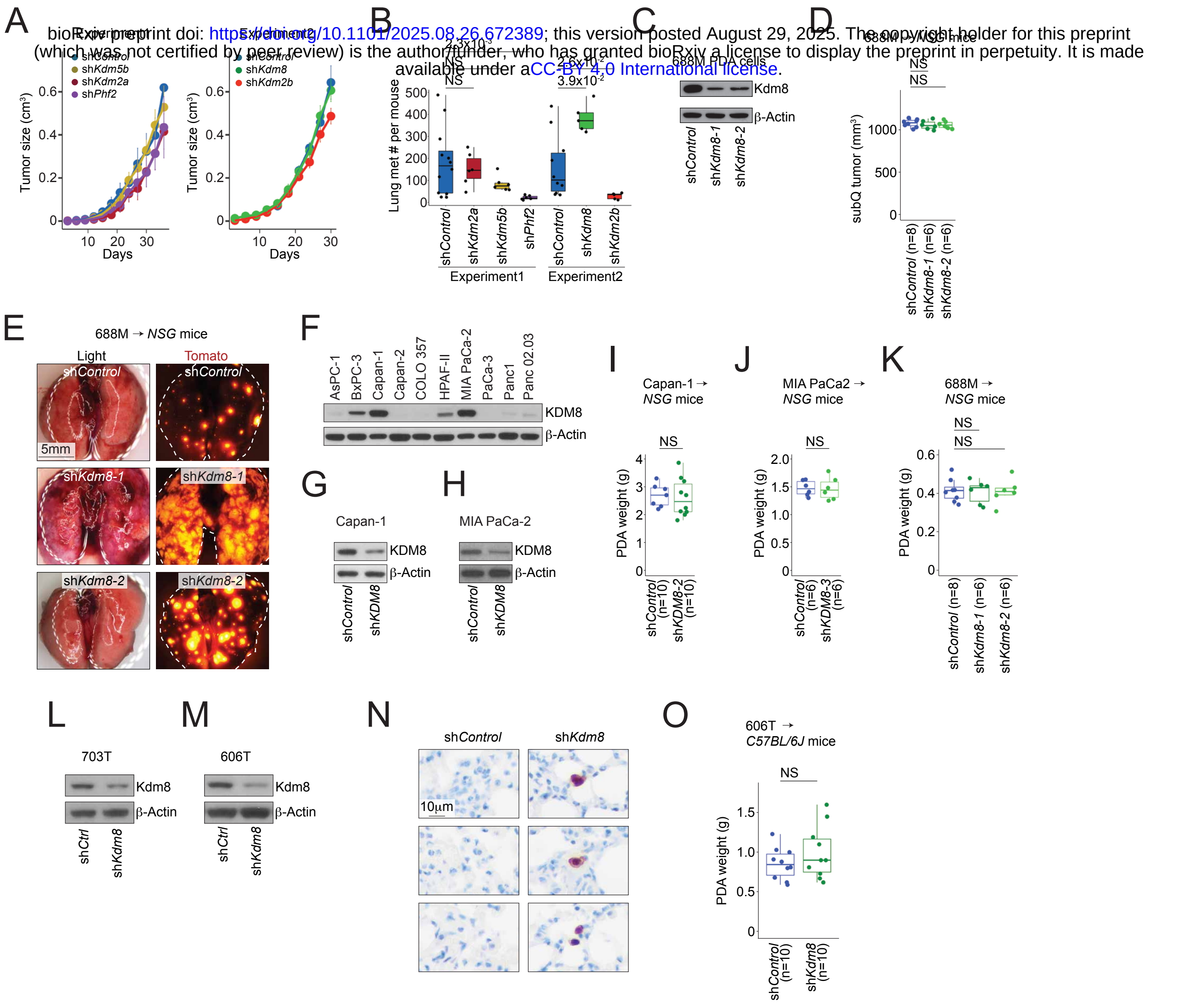

Figure S1, related to Figure 1.
(A,B) Subcutaneous tumor growth (A) and counts of lung metastases (B) seeded from the subcutaneous tumors in the *NSG* mice receiving the Tomato-positive 688M PDA cells transduced with control (sh*Control*, experiment 1, n=12; experiment 2, n=10) or shRNA targeting the indicated *Kdm* genes (sh*Kdm2a*, n=6; sh*Kdm5b*, n=6; sh*Phf2*, n=6; sh*Kdm8*, n=5; sh*Kdm2b*, n=5). Two independent experiments are shown. (C) *Kdm8* knockdown in 688M cells using two independent shRNAs. (D,E) Subcutaneous tumor size (D) and representative light (left) and fluorescent (right) images of the lungs (E) upon sacrifice for the tumor studies in Figure S1B. (F) Immunoblot for the endogenous abundance of KDM8 and β-Actin in the indicated human PDA cell lines. (G,H) *KDM8* knockdown in Capan-1 (G) and MIA PaCa-2 (H) human PDA cells using a *KDM8*-targeting shRNA. (I-K) The primary PDA tumor weight for the orthotopic tumor studies using Capan-1, MIA PaCa-2, and 688M PDA cells. (L,M) *Kdm8* knockdown in 703T (L) and 606T (M) murine PDA cells using a *Kdm8*-targeting shRNA. (N) Representative Tomato IHC images demonstrating individual micrometastatic PDA cells in the lungs of the *B6* mice receiving sh*Kdm8*-expressing 606T PDA cells (right) compared to control (left). (O) The primary PDA tumor weight in each *B6* mouse orthotopically transplanted with 606T PDA cells as in Figures 1N-1P. NS, not significant. Each dot is a mouse and boxes represent medians with interquartile range between 25th and 75th percentiles.

Figure S2

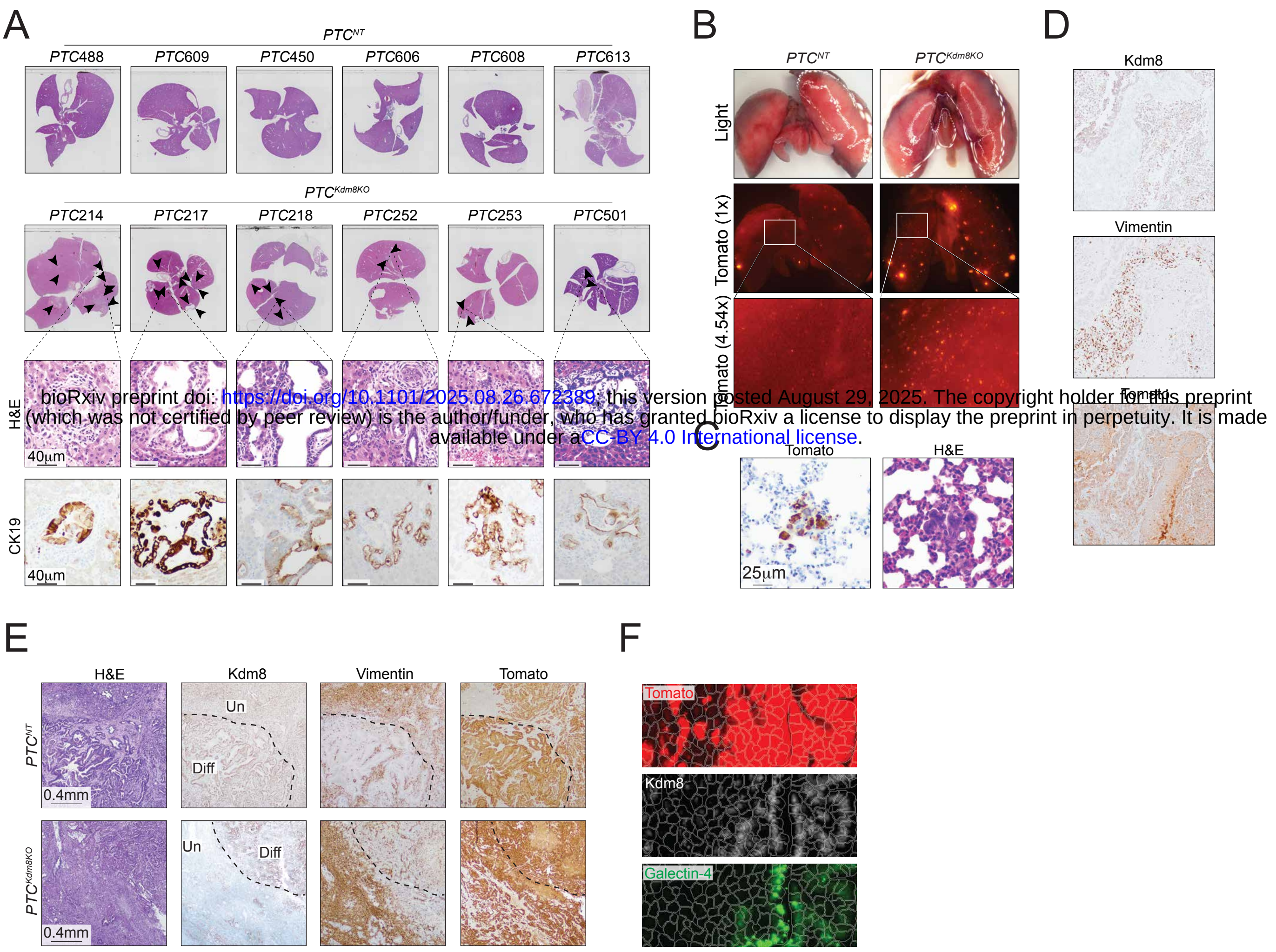

bioRxiv preprint doi: https://doi.org/10.1101/2025.08.26.672389; this version posted August 29, 2025. The copyright holder for this preprint (which was not certified by peer review) is the author/funder, who has granted bioRxiv a license to display the preprint in perpetuity. It is made available under aCC-BY 4.0 International license.

Figure S2, related to Figure 2.
(A) Haematoxylin and eosin staining (H&E) and CK19 immunohistochemistry (IHC) of liver samples from PTC^{NT} (top row) and PTC^{Kdm8KO} (2nd, 3rd, and 4th rows) mice. (B) Representative light (top) and fluorescent images of the whole lungs (2nd row) and zoom-in regions (3rd row) from a PTC^{NT} (left) and a PTC^{Kdm8KO} (right) mouse. (C) Representative H&E (right) and Tomato IHC (left) of lung micrometastases in a PTC^{Kdm8KO} mouse. (D) Representative IHC of Kdm8, Vimentin, and Tomato of serial sections from a primary PDA region as in Figure 2M. (E) Representative H&E and IHC of Kdm8, Vimentin, and Tomato of serial sections from primary PDA tumors of a PTC^{NT} (top) and a PTC^{Kdm8KO} (bottom) mouse. Dotted lines demarcate the fully differentiated (diff) and poorly differentiated (Un) regions. (F) Representative images as in Figure 2I of a PTC^{Kdm8KO} tumor with automated cell segmentation, overlaid with pseudocolors for Tomato, Kdm8, and Galectin-4 (top to bottom).

Figure S3

bioRxiv preprint doi: https://doi.org/10.1101/2025.08.26.672389; this version posted August 29, 2025. The copyright holder for this preprint (which was not certified by peer review) is the author/funder, who has granted bioRxiv a license to display the preprint in perpetuity. It is made available under aCC-BY 4.0 International license.

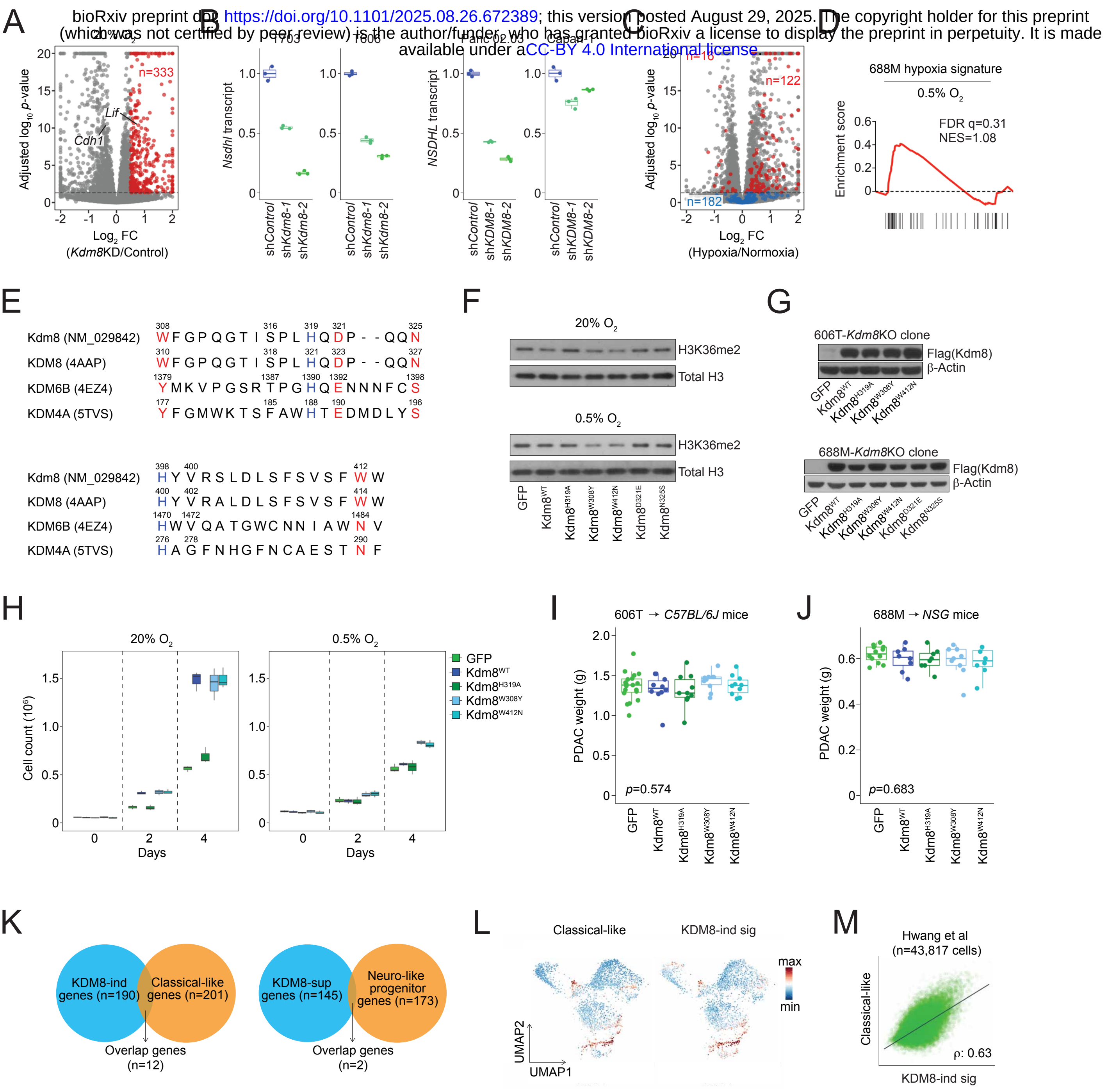

Figure S3, related to Figure 3.

(A) Volcano plot of differential genes induced upon *Kdm8* knockdown in normoxia (n=333 genes upregulated, red). *Lif* and *Cdh1* are highlighted for Figure 3F. (B) Expression of *Nsdhl* and *NSDHL* following *Kdm8* and *KDM8* knockdown the indicated PDA cell lines, as measured by quantitative PCR. (C) Volcano plot of differential genes induced or suppressed by hypoxia in 688M cells. Genes that are induced by *Kdm8* knockdown are highlighted (red, adjusted p-value < 0.05; blue, non-significant under hypoxia). (D) GSEA of the 688M hypoxia gene signature enriched in *Kdm8* knockdown 688M cells cultured in hypoxia. (E) Alignment of the JmjC domains of human KDM8, KDM6B, and KDM4A (with PDB identifiers). Red, non-conserved residues that share consensus in KDM6B and KDM4A; blue, conserved residues among all three KDMs. Murine Kdm8 is shown for comparison. KDM8 residue 328-399 are omitted due to the absence of conserved residues in KDM6B and KDM4A, and their high divergence within KDM8. (F) Immunoblots of H3K36me2 and total H3K36 in *Kdm8* knockout 688M cells re-expressing the indicated Kdm8 variants cultured in normoxia (top) or hypoxia (bottom). (G) Immunoblots of indicated Flag tagged Kdm8 variants in *Kdm8*-knockout 606T (top) and 688M (bottom) clones. β-actin shows equal loading. (H) Cell counts of *Kdm8* knockout 688M cells re-expressing the indicated Kdm8 variants cultured in normoxia (left) or hypoxia (right) following 0, 2, and 4 days post seeding. Comparable results were observed using 606T cells (not shown). (I,J) Pancreatic tumor weights at the time of sacrifice for the tumor studies shown in Figures 3E and 3F, respectively. (K) Venn diagrams demonstrating the shared genes between the KDM8-indued and the classical gene signatures and those between the KDM8-suppressed and the neuro-like progenitor (Hwang et al, 2022 Nat Genet) gene signatures. (L,M) UMAP demonstrating human PDA malignant cells defined in Hwang et al, 2022 Nat Genet colored by the KDM8-induced and classical gene signatures (L). The relationship between the scores of the two gene signatures is shown with the Pearson correlation coefficient ρ (M).

bioRxiv preprint doi: https://doi.org/10.1101/2025.08.26.672389; this version posted August 29, 2025. The copyright holder for this preprint (which was not certified by peer review) is the author/funder, who has granted bioRxiv a license to display the preprint in perpetuity. It is made available under aCC-BY 4.0 International license.

Figure S4

bioRxiv preprint doi: https://doi.org/10.1101/2025.08.26.672389; this version posted August 29, 2025. The copyright holder for this preprint (which was not certified by peer review) is the author/funder, who has granted bioRxiv a license to display the preprint in perpetuity. It is made available under aCC-BY 4.0 International license.

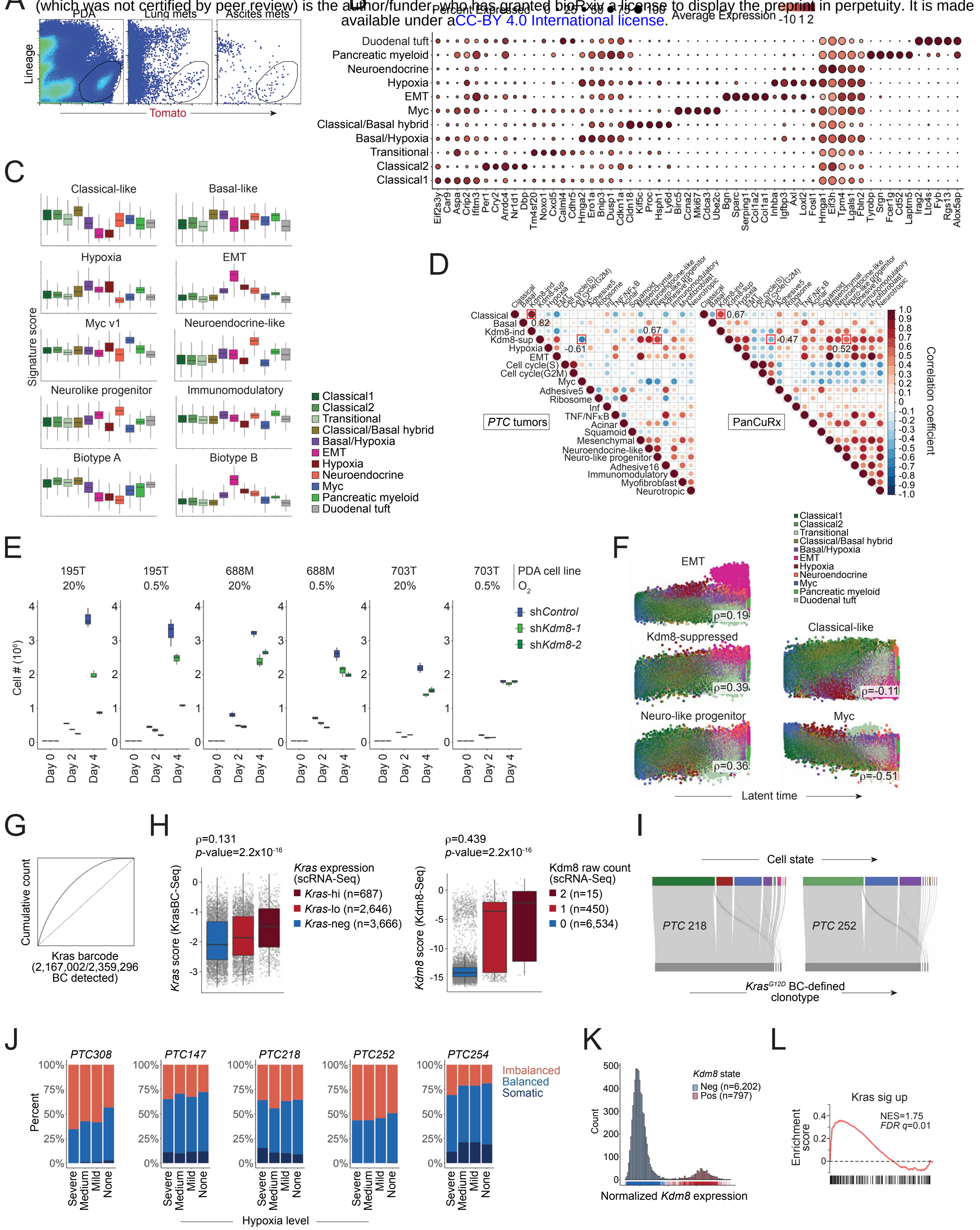

bioRxiv preprint doi: https://doi.org/10.1101/2025.08.26.672389; this version posted August 29, 2025. The copyright holder for this preprint (which was not certified by peer review) is the author/funder, who has granted bioRxiv a license to display the preprint in perpetuity. It is made available under a CC-BY 4.0 International license.

Figure S4, related to Figure 4.

(A) Representative FACS density plots of the Tomato-positive, lineage (Cd31, Cd45, Ter119, and F4/80)-negative malignant cells from the primary PDA tumor, lung metastases, and ascites of a PTC^{Kdm8KO} mouse. The gate used to sort the Tomato+lineage- cells is shown. (B) Dot plot showing select differential genes across the identified Seurat cell clusters defined in Figure 4B. Dot size represents the percentage of cells expressing the differential genes and color represents the abundance of the differential genes. (C) Relative abundance of the previously reported malignant programs (Hwang et al, 2022 Nat Genet; Di Chiaro, et al, 2024 Cancer Cell; Moffitt et al, 2015 Nat Genet) and GSEA hallmark gene signatures across the 11 clusters of malignant cells defined in Figure 4B. (D) Dot plots demonstrating the Pearson correlation coefficient between any two malignant programs as in C quantified in the current study (left) and the bulk tumors from the PanCuRx PDA cohort (right). Select comparisons are highlighted with their Pearson correlation coefficients annotated. (E) Total cell counts on the indicated days following normoxic or hypoxic culture of 3 murine PDA cell lines expressing control or 2 *Kdm8*-targeting shRNAs. (F) Abundance of the indicated malignant programs plotted along the latent time and colored by the clusters defined in Figure 4B. Pearson correlation coefficients (ρ) are shown. (G) Cumulative plot for the AAV plasmid library. (H) Relationships between the transcript abundance measure by 10x scRNA-Seq (x-axis) and KrasBC-Seq (left, y-axis) or Kdm8-Seq (right, y-axis). Pearson correlation coefficients (ρ) and p-values are shown. (I) Sankey diagrams for all barcoded (bottom) malignant cells isolated from two PTC^{Kdm8KO} mice that correspond to the malignant programs defined in Figure 4B (top). (J) Proportions of malignant cells isolated from 5 PTC^{Kdm8KO} tumors, categorized by the indicated $Kras^{G12D}/Kras^{WT}$ allelic conformation, across varying levels of hypoxia (x-axis, from none to severe), quantified using the GSVA method. (K) Histogram of *Kdm8* expression in the malignant cells from 5 PTC^{Kdm8KO} tumors (n=6,999) estimated by Kdm8-Seq. (L) GSEA for the indicated hallmark gene signature (Kras signaling up) enriched in $Kdm8^{neg}$ malignant cells as in Figure 4L.

bioRxiv preprint doi: https://doi.org/10.1101/2025.08.26.672389; this version posted August 29, 2025. The copyright holder for this preprint (which was not certified by peer review) is the author/funder, who has granted bioRxiv a license to display the preprint in perpetuity. It is made available under aCC-BY 4.0 International license.

A

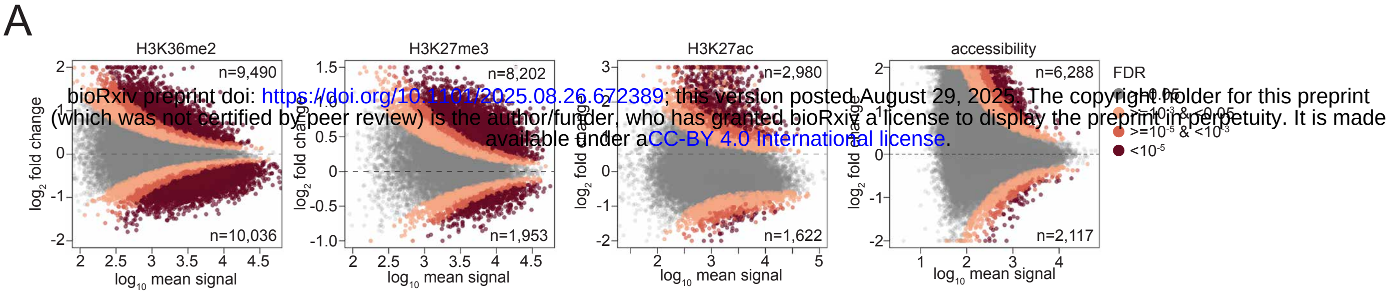

B

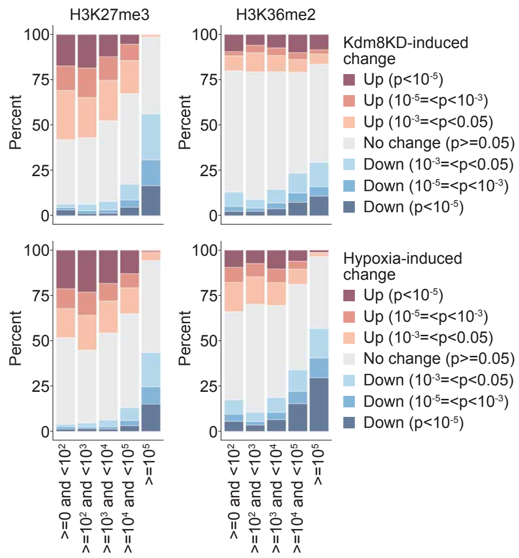

C

	H3K36me2		H3K27me3	
Dis to TSS	Hypoxia-suppressed	*Kdm8*KD-suppressed	Hypoxia-suppressed	*Kdm8*KD-suppressed
Proximal (<10^3 bp)	383/3,447 (11.1%)	320/3,447 (9.3%)	59/1,292 (4.6%)	82/1,292 (6.3%)
Distal (>=10^3 bp)	9,653/35,228 (27.4%)	6,820/35,228 (19.4%)	1,894/18,717 (10.1%)	2,468/18,717 (13.2%)
p-value	4.06×10^{-96}	7.91×10^{-48}	1.08×10^{-10}	1.37×10^{-12}
Dis to TSS	Hypoxia-induced	*Kdm8*KD-induced	Hypoxia-induced	*Kdm8*KD-induced
Proximal (<10^3 bp)	1,040/3,447 (30.2%)	712/3,447 (20.7%)	703/1,292 (54.4%)	738/1,292 (57.1%)
Distal (>=10^3 bp)	8,450/35,228 (24.0%)	7,240/35,228 (20.6%)	7,496/18,717 (40.0%)	7,439/18,717 (39.7%)
p-value	9.54×10^{-16}	0.9	4.34×10^{-24}	1.50×10^{-34}

D

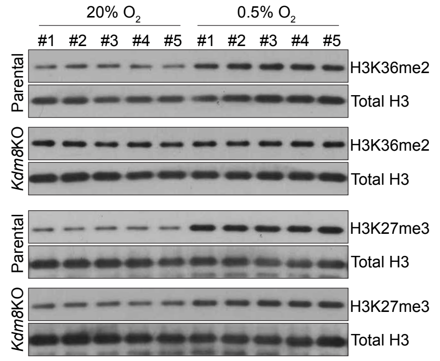

E

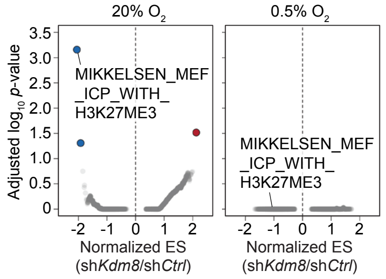

F

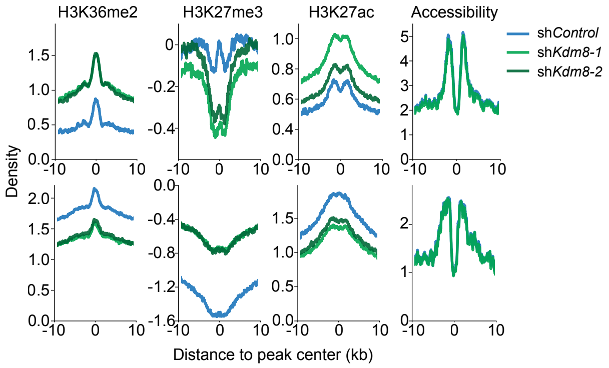

G

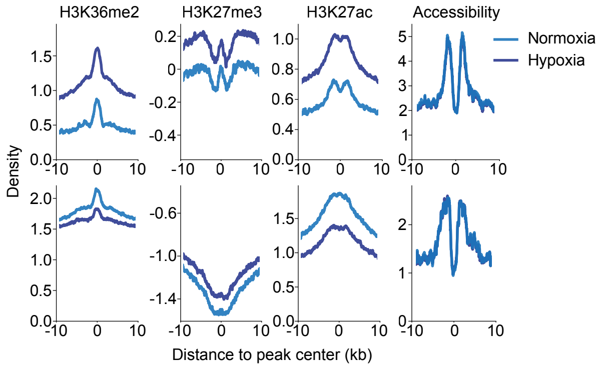

bioRxiv preprint doi: https://doi.org/10.1101/2025.08.26.672389; this version posted August 29, 2025. The copyright holder for this preprint (which was not certified by peer review) is the author/funder, who has granted bioRxiv a license to display the preprint in perpetuity. It is made available under aCC-BY 4.0 International license.

Figure S5, related to Figure 5.

(A) Hypoxia-induced differential H3K36me2, H3K27me3, and H3K27ac ChIP-Seq signals, as well as chromatin accessibility measured by ATAC-Seq (left to right), plotted against the mean read counts per region, as in Figure 5A. Dotted lines represent zero log_2 fold change (FC), with positive values representing hypoxia-induced increases. FDR (false discovery rate) reflects the probability that a differential region exhibits a significant change induced by hypoxia. The number of significantly altered regions (FDR < 0.05) is indicated at the top and bottom of each plot. (B) Annotations of all H3K36me2 (right) and H3K27me3 (left) regions by Homer's annotatePeaks.pl, based on their distance to the nearest transcription start site (TSS), grouped into 5 bins with indicated genomic ranges in base pairs (x-axis). The y-axis shows the proportion of regions within each genomic range, stratified by the p-value of *Kdm8* knockdown (*Kdm8*KD)-induced (top) and hypoxia-induced (bottom) changes. (C) Fractions of proximal (<10^3 bp to TSS) and distal (>=10^3 bp to TSS) H3K36me2 (left) and H3K27me3 (right) genomic regions that are induced (bottom) or suppressed (top) by hypoxia or *Kdm8*KD. Chi-squared test p-values for the association between distance to TSS and *Kdm8*KD- or hypoxia-induced changes are shown. (D) Immunoblots showing H3K36me2, H3K27me3, and total H3 levels in parental 688M and a *Kdm8* knockout (*Kdm8*KO) clone cultured in normoxia or hypoxia (0.5% O_2). Data from 5 technical replicates per group are shown. (E) Gene set enrichment analysis (GSEA) of the curated gene sets (C2, v7.5.1) depleted in *Kdm8* knockdown 688M cells cultured in normoxia (left) or hypoxia (right). Normalized enrichment score (ES) for the gene signatures and the corresponding FDR-adjusted p-values are shown. sh*Ctrl*, control shRNA. The MEF cell derived gene signature bearing the H3K27me3 mark (Mikkelsen *et al*, 2008 Nature) is highlighted. (F,G) Aggregate signals of H3K36me2, H3K27me3, and H3K27ac ChIP-Seq, as well as chromatin accessibility measured by ATAC-Seq, within 3,973 genomic regions that are co-induced by *Kdm8*KD and hypoxia for H3K36me2 (top), and 3,838 regions that are co-suppressed by *Kdm8*KD and hypoxia for H3K36me2 (bottom), in control or sh*Kdm8*-expressing cells (F), and in 688M cells cultured in normoxia or hypoxia (G).

bioRxiv preprint doi: https://doi.org/10.1101/2025.08.26.672389; this version posted August 29, 2025. The copyright holder for this preprint (which was not certified by peer review) is the author/funder, who has granted bioRxiv a license to display the preprint in perpetuity. It is made available under aCC-BY 4.0 International license.

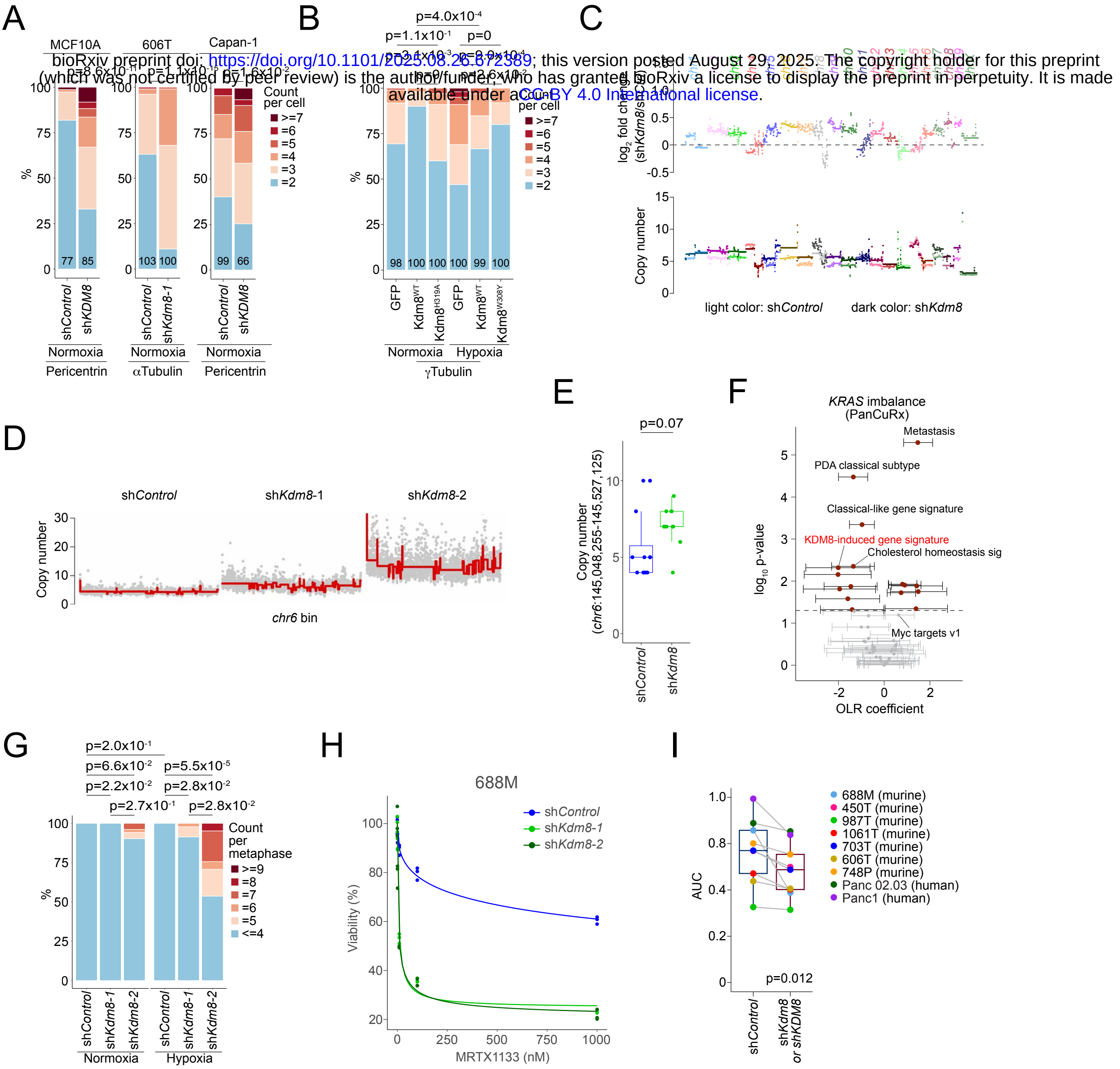

bioRxiv preprint doi: https://doi.org/10.1101/2025.08.26.672389; this version posted August 29, 2025. The copyright holder for this preprint (which was not certified by peer review) is the author/funder, who has granted bioRxiv a license to display the preprint in perpetuity. It is made available under aCC-BY 4.0 International license.

Figure S6, related to Figure 6.

(A) Quantifications of anaphase polarities by Pericentrin or αTubulin immunofluorescence (IF) in indicated control and *Kdm8*/*KDM8* deficient PDA cells as in Figure 6E. Numbers in the plot indicate the number of cells quantified in each group. (B) Proportions of *Kdm8* knockout 688M cells re-expressing indicated Kdm8 variants cultured in normoxia or hypoxia with indicated anaphase polarities quantified by γTubulin IF. Numbers in the plot indicate the number of cells quantified in each group. (C) Single-cell whole genome sequencing (scWGS) showing $\log_2$ fold change (sh*Kdm8*/sh*Control*, top) and the copy number of genomes in control (n=12) and sh*Kdm8*-expressing 688M cells (n=11, bottom). (D) Bulk whole genome sequencing showing chromosome 6 copy-number alterations in control and 688M cells expressing two sh*Kdm8*s. (E) Copy numbers of the indicated genomic location on chromosome 6 (mm39) where *Kras* is located in control and sh*Kdm8*-expressing 688M cells using scWGS as in (C). p-value = 0.07 using two-sided Wilcoxon test. (F) Ordinal logistic regression (OLR) for the association between the *KRAS* allelic imbalance (wt, 0; balanced, 1; minor, 2; major, 3) and the GSEA hallmark transcriptomic programs, anatomic location (primary site, 0; liver metastasis, 1), PDA subtype (classical, 1; basal, 0), and KDM8-induced gene signature in the PanCuRx PDA cohort. Coefficients with the 2.5/97.5% confidence intervals (x-axis) and $\log_{10}$ converted p-values (y-axis) are shown. KDM8-induced gene signature is highlighted. Dotted line represents statistical significance (p=0.05). (G) Quantifications of FISH as in Figure 6I using a chromosome 18 centromeric probe (yellow, Figure 6H) in control and sh*Kdm8*-expressing 688M cells cultured in normoxia and hypoxia. Proportions of control and the sh*Kdm8* metaphase 688M cells cultured in normoxia or hypoxia are shown. (H) Percent viability of 688M cells expressing control or *Kdm8*-targeting shRNAs treated with indicated concentrations of $Kras^{G12D}$ inhibitor MRTX1133. Results from three repeated experiments are shown. (I) Boxplot showing responses to MRTX1133 quantified by the area under the dose-response curve (AUC) for the indicated PDA cell lines. Significance was determined via paired student t-test following a Shapiro-Wilk test for normality.

Table S1. Survival and clinical features of disease progression in the PDA GEM model

Date of birth	Date of surgery	Date of death	Days	Surgical Procedure	Animal ID	Mouse genotype	Virus infused	Gender	Infused volume (uL)	PDA tumor	Splenomegaly	Ascites	Lung met #	Liver met #	Peritoneal met
3/17/22	5/6/22	2/16/23	286	Pancreatic ductal injection	147	PTC	AAV-U6-sgKdm8(J1)-KrasG12D(BC)	F	80	Y	Y	Y	26	0	Y
3/17/22	5/6/22	11/19/22	197	Pancreatic ductal injection	305	PTC	AAV-U6-sgKdm8(J1)-KrasG12D(BC)	F	110	N	NA	NA	NA	NA	NA
3/17/22	5/11/22	2/27/23	292	Pancreatic ductal injection	306	PTC	AAV-U6-sgKdm8(J1)-KrasG12D(BC)	M	10	N	NA	NA	NA	NA	NA
3/17/22	5/11/22	2/27/23	292	Pancreatic ductal injection	142	PTC	AAV-U6-sgKdm8(J1)-KrasG12D(BC)	M	100	N	NA	NA	NA	NA	NA
3/17/22	5/11/22	2/28/23	293	Pancreatic ductal injection	307	PTC	AAV-U6-sgKdm8(J1)-KrasG12D(BC)	F	70	Y	**Y**	Y	47	0	Y
3/24/22	5/11/22	11/4/22	177	Pancreatic ductal injection	308	PTC	AAV-U6-sgKdm8(J1)-KrasG12D(BC)	M	80	Y	**Y**	Y	8	NA	N
5/23/22	6/29/22	2/8/23	224	Pancreatic ductal injection	214	PTC	AAV-U6-sgKdm8(J1)-KrasG12D(BC)	M	100	Y	**Y**	Y	109	12	Y
5/23/22	6/29/22	10/27/22	120	Pancreatic ductal injection	215	PTC	AAV-U6-sgKdm8(J1)-KrasG12D(BC)	M	90	N	NA	NA	NA	NA	NA
5/23/22	6/29/22	12/9/22	163	Pancreatic ductal injection	216	PTC	AAV-U6-sgKdm8(J1)-KrasG12D(BC)	M	90	N	NA	NA	NA	NA	NA
5/23/22	6/29/22	1/2/23	187	Pancreatic ductal injection	217	PTC	AAV-U6-sgKdm8(J1)-KrasG12D(BC)	M	100	Y	N	Y	51	18	Y
5/23/22	6/29/22	1/27/23	212	Pancreatic ductal injection	218	PTC	AAV-U6-sgKdm8(J1)-KrasG12D(BC)	M	120	Y	**Y**	Y	39	4	Y
5/23/22	6/29/22	10/21/22	114	Pancreatic ductal injection	219	PTC	AAV-U6-sgKdm8(J1)-KrasG12D(BC)	M	130	N	NA	NA	NA	NA	NA
8/29/22	10/6/22	1/24/23	110	Pancreatic ductal injection	252	PTC	AAV-U6-sgKdm8(J1)-KrasG12D(BC)	F	50	Y	**Y**	Y	60	2	Y
8/29/22	10/6/22	2/18/23	135	Pancreatic ductal injection	253	PTC	AAV-U6-sgKdm8(J1)-KrasG12D(BC)	M	50	Y	**Y**	Y	25	1	Y
8/29/22	10/6/22	2/3/23	120	Pancreatic ductal injection	254	PTC	AAV-U6-sgKdm8(J1)-KrasG12D(BC)	M	50	Y	**Y**	N	20	0	Y
1/12/23	2/22/23	7/17/23	145	Pancreatic ductal injection	378	PTC	AAV-U6-sgKdm8(J1)-KrasG12D(BC)	M	100	N	NA	NA	NA	NA	NA
1/12/23	2/22/23	7/17/23	145	Pancreatic ductal injection	379	PTC	AAV-U6-sgKdm8(J1)-KrasG12D(BC)	M	50	N	NA	NA	NA	NA	NA
1/12/23	2/22/23	6/21/23	119	Pancreatic ductal injection	501	PTC	AAV-U6-sgKdm8(J1)-KrasG12D(BC)	M	50	Y	N	Y	32	2	Y
3/28/23	5/22/23	4/4/24	318	Pancreatic ductal injection	449	PTC	AAV-U6-sgNT-KrasG12D	M	40	Y	NA	N	6	0	N
3/28/23	5/22/23	12/5/23	197	Pancreatic ductal injection	450	PTC	AAV-U6-sgNT-KrasG12D	M	280	Y	NA	Y	8	0	N
4/3/23	5/22/23	11/17/23	179	Pancreatic ductal injection	488	PTC	AAV-U6-sgNT-KrasG12D	M	80	Y	Y	Y	15	0	Y
4/3/23	5/22/23	2/15/24	269	Pancreatic ductal injection	491	PTC	AAV-U6-sgNT-KrasG12D	M	30	Y	NA	N	18	0	N
4/22/23	5/22/23	12/26/23	218	Pancreatic ductal injection	606	PTC	AAV-U6-sgNT-KrasG12D	M	70	Y	Y	Y	7	0	N
4/22/23	5/22/23	1/2/24	225	Pancreatic ductal injection	608	PTC	AAV-U6-sgNT-KrasG12D	M	60	Y	Y	Y	24	0	N
4/22/23	5/22/23	11/28/23	190	Pancreatic ductal injection	609	PTC	AAV-U6-sgNT-KrasG12D	M	120	Y	Y	Y	12	0	N
4/22/23	5/22/23	4/4/24	318	Pancreatic ductal injection	612	PTC	AAV-U6-sgNT-KrasG12D	M	25	Y	Y	Y	88	0	N
4/22/23	5/22/23	11/7/23	169	Pancreatic ductal injection	613	PTC	AAV-U6-sgNT-KrasG12D	M	40	Y	NA	NA	NA	NA	NA
10/24/24	12/10/24	4/23/25	134	Pancreatic ductal injection	2445	PTC	AAV-U6-sgNT-KrasG12D	F	20	Y	Y	Y	18	0	Y
10/24/24	12/10/24	4/25/25	136	Pancreatic ductal injection	2446	PTC	AAV-U6-sgNT-KrasG12D	F	50	Y	Y	Y	22	0	N
10/30/24	12/10/24	4/23/25	134	Pancreatic ductal injection	2429	PTC	AAV-U6-sgNT-KrasG12D	F	50	Y	Y	Y	1	0	N
10/31/24	12/10/24	4/7/25	118	Pancreatic ductal injection	2487	PTC	AAV-U6-sgNT-KrasG12D	F	70	Y	Y	N	77	0	N
